# Fine Morphology of the Mouthparts in *Cheilocapsus nigrescens* (Hemiptera: Heteroptera: Miridae) Reflects Adaptation for Phytophagous Habits

**DOI:** 10.3390/insects10050143

**Published:** 2019-05-19

**Authors:** Yan Wang, Lingfei Li, Wu Dai

**Affiliations:** Key Laboratory of Plant Protection Resources and Pest Management of the Ministry of Education, College of Plant Protection, Northwest A&F University, Yangling 712100, Shaanxi, China; wangyan105422@163.com (Y.W.); m19801315617@163.com (L.L.)

**Keywords:** phytophagy, stylet, sensilla, fine morphology, scanning electron microscopy

## Abstract

To obtain a better understanding of feeding adaptations, the fine structure of the mouthparts in adults of *Cheilocapsus nigrescens* Liu and Wang, including the sculpture and interlocking mechanisms of the stylets and distribution and abundance of sensilla located on the labium, were studied using scanning electron microscopy. The mouthparts are similar to those of previously studied mirid species in most aspects and composed of a cone-shaped labrum, a tube-like, four-segmented labium with a deep groove on the anterior side, and a stylet fascicle consisting of two mandibular and two maxillary stylets. Each mandibular stylet tip has about 6–8 indistinctive notches, which help in penetrating the leaf surface. A series of transverse squamous textures are present on the adaxial surface of the mandibular stylets. The maxillary stylets interlock to form a food canal and a salivary canal, equipped with an external longitudinal process that engages grooves in the mandibular stylets. Three kinds of sensilla, including four types of sensilla basiconica (I, II, III, and IV), four types of sensilla trichodea (I, II, III, and IV), and one type of sensillum campaniformium, occur at different locations on the labium. Among them, sensilla trichodea I and II are the most abundant; sensilla basiconica II occurs between the first segment and second segment, and between the third and fourth segment. The tripartite apex of the labium consists of two lateral lobes and an apical plate. Each lateral lobe possesses a field of 11 sensilla basiconica IV and 1 sensillum trichodeum IV. The morphology of the mouthparts and the distribution of sensilla located on the labium in *C. nigrescens* are discussed with respect to their possible taxonomic and functional significance. In particular, the indistinct notches of the mandibular stylet and smooth inner surface of the right maxillary stylets are suited primarily for phytophagy.

## 1. Introduction

Insect mouthparts are among the most important feeding structures, and one of the most intensively examined structures among animals [1]. The complexity and variety in the structures of the mouthparts reflect the great variety in feeding habits and strategies employed by different insects, resulting from evolutionary diversification over hundreds of millions of years [2]. Mouthpart structure in insects remains closely tied to their feeding habits and hosts. Thus, the study of the functional morphology and variation among mouthparts is important for understanding feeding habits and the evolution of mouthparts of insects [3,4,5]. Such study is needed not only to reveal patterns in mouthpart evolution [6] but also to better understand the economic consequences of insect feeding, particularly feeding injury caused to agricultural plants, crop pollination, and transmission of insect-vectored diseases [1].

True bugs (Hemiptera: Heteroptera) exhibit a wide variety of feeding behaviors and preferences, ranging from predation and ectoparasitism on vertebrate and invertebrate animals to feeding on various plant parts and tissues (e.g., vascular fluids, cell contents, and seeds). Previous studies have indicated that this group exhibits considerable variation in mouthpart morphology. Extensive data are available on some aspects of mouthpart morphology of Heteroptera based on light and scanning electron microscopy [7,8,9,10,11,12,13,14,15,16,17], but few studies have examined details of mouthpart fine structure. The family Miridae (plant bugs) is the most diverse and one of the most economically important groups in Heteroptera [18], containing more than 11,020 valid described species [19], including many important agricultural pests as well as predators that can be used as biological control agents [18,20]. Plant bugs exhibit a wide range of food preferences and behaviors, including phytophagy, carnivory, and omnivory [20]. Feeding preferences in the Miridae probably are more diverse than in any other heteropteran family [18]. Correspondingly, feeding on different plants or prey requires specialization of mouthparts, digestive tracts, enzyme complexes, and biochemical pathways [21,22]. Mirid mouthparts perform crucial functions in their feeding, with the sensillar equipment fine-tuned by strong selection [10,11,23,24,25,26,27]. Clarification of the fine morphology and sensory mechanisms of mirid mouthparts can help to elucidate their ecology and biology, especially trophic niches. Nevertheless, the mouthparts of mirid species have received only sporadic study. Previous studies on mouthparts in Miridae have concentrated mostly on terminal labial sensilla [23,24,25], the interlocking mechanisms of maxillae and mandibles [13,26], and the gross morphology of the mandible and maxilla [7,10,11,13,26,27,28]. More detailed investigations with expanded taxonomic sampling are needed to determine how much variability occurs in these structures and how such variation may relate to differences in feeding behaviors and preferences.

Phytophagous mirids often exploit nutrient-rich mesophyll and apical meristems, including new foliage, flower buds, flowers, pollen, and the developing seeds of their hosts [18]. However, for these insects, little is known about the finer aspects of the mouthpart structure and their role in locating feeding sites on the host plant. The plant bug *Cheilocapsus nigrescens* Liu and Wang is a common phytophagous species distributed in Gansu, Fujian, Sichuan, Shaanxi, Hubei, and Yunnan Provinces, China [29,30]. The fine structure of the mouthparts of *C. nigrescens* and the function of these mouthpart structures in feeding have not been studies previously.

The aim of this study is to provide the first detailed fine morphological characterization of the mouthparts of *C. nigrescens* using scanning electron microscope (SEM). The fine structure, location, and distribution of different sensilla types on mouthparts are investigated, and the possible functions of these sensilla are discussed. This study provides more data for future comparative morphological studies between phytophagous and zoophagous mirids.

## 2. Materials and Methods 

### 2.1. Insect Collecting

Adults of *C. nigrescens* used for SEM in this study were collected at the Huoditang field station of Northwest A&F University in Ningshan County, Shaanxi Province, China (33°43′N, 108°45′E, elev. 1580 m) in August 2016. Specimens were preserved in 95% ethanol and stored at 4 °C.

### 2.2. Samples for SEM

Adults were placed in 95% ethanol. Their heads were removed with dissecting needles under a stereomicroscope (Olympus SZX10, Tokyo, Japan) and then dehydrated in 100% ethanol two times for 30 min. transferred to a graded series of tert-butyl ethanol (TBA) solutions of 25%, 50%, and 75% (ethanol: TBA was 3:1; 1:1; 1:3) for 15 min at each concentration, and finally 100% TBA for 30 min. The samples were then placed into a freeze-drier (VFD-21S, SHINKKU VD, Tokyo, Japan) for 3h. The dried specimens were mounted on aluminum stubs using double-sided copper sticktape and coated with gold/palladium (40/60) in a high resolution sputter coater (MSP-1S, SHINKKU VD, Tokyo, Japan), and then observations were performed with a T-3400 SEM (Hitachi, Tokyo, Japan) operated at 15 kV or Nova Nano SEM-450 (FEI, Hillsboro, OR, USA) at 5–10 kV. Thirty individuals of this species were observed.

### 2.3. Image Processing and Morphometric Measurement

Photographs were observed and measured after being imported into Adobe Photoshop CS6 (Adobe Systems, San Jose, CA, USA). Statistical analyses were executed using SPSS 19.0 (SPSS, Chicago, IL, USA). Graphs were fitted by using Microsoft Office Excel 2007.

### 2.4. Terminology

For classification of sensilla, the systems of Shields [31] were used in addition to the more specialized nomenclature from other studies [8,32,33,34]. The terminology of mouthparts is adapted from that of Spangenberg et al. [35].

## 3. Results

### 3.1. Gross Morphology of the Mouthparts

**Figure 1:**
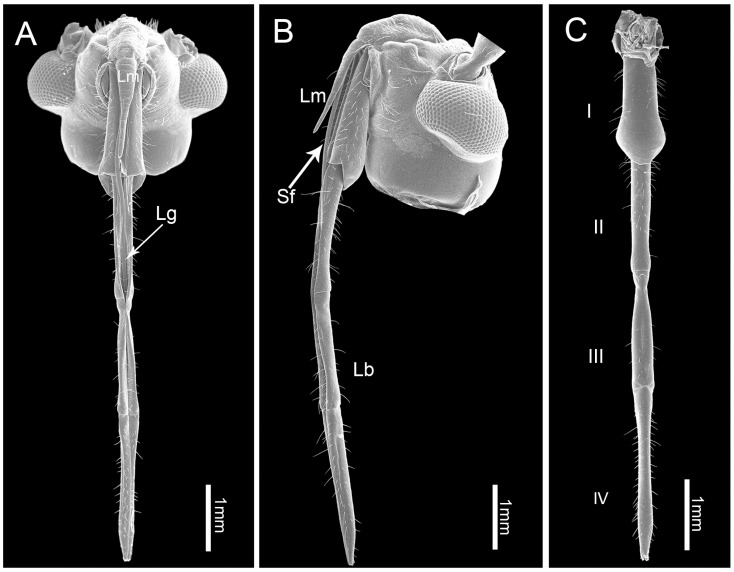
Scanning electron micrographs of the head of adult *C. nigrescens*. (**A**) Dorsal view; (**B**) lateral view; and (**C**) ventral view showing four-segmented labium (I–IV). Lb, labium; Lg, labial groove; Lm, labrum; and Sf, stylet fascicle.

As in other heteropterans, the mouthparts of *C. nigrescens* arise from the anterior part of the head capsule and consist of a relatively small labrum (Lm) and a tubular four-segmented labium (Lb) with a deep longitudinal labial groove (Lg) on the anterior surface that houses the stylet fascicle consisting of two mandibular and two maxillary stylets. The triangular labrum covers the proximal end of the first labial segment (Figure 1A). Various types of sensilla are symmetrically distributed on either sides of the labial groove or positioned on the distal end of the labium. No obvious differences were noted between the mouthpart structure of females and males except for their length. The total length in females is 3798.4 ± 37.8 μm (*n =* 3), and for males is 3574.0 ± 140.7 μm (*n =* 12) (Table 1).

### 3.2. Labrum

**Figure 2:**
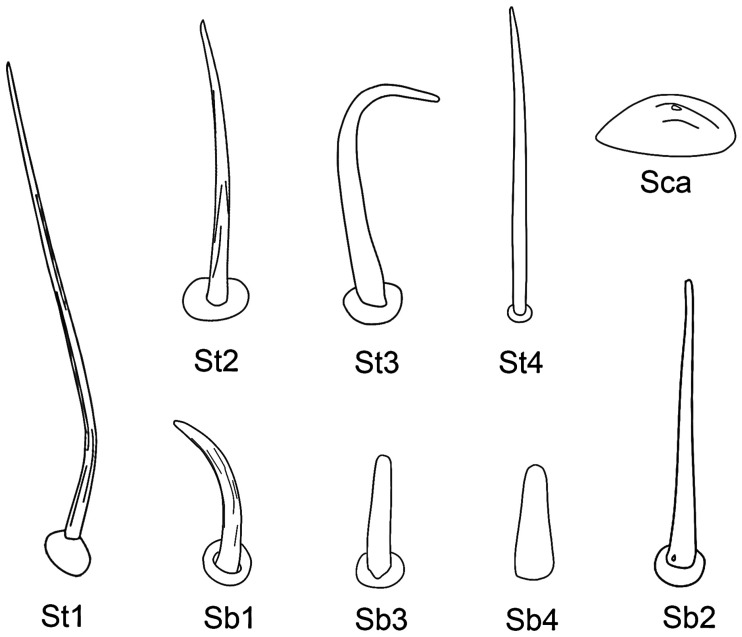
Diagrams of different types of sensilla on mouthparts of *C. nigrescens*. Sb1, sensilla basiconica I; Sb2, sensilla basiconica II; Sb3, sensilla basiconica III; Sb4, sensilla basiconica IV; Sca, sensilla campaniformia; St1, sensilla trichodea I; St2, sensilla trichodea II; St3, sensilla trichodea III; and St4, sensilla trichodea IV.

**Figure 3:**
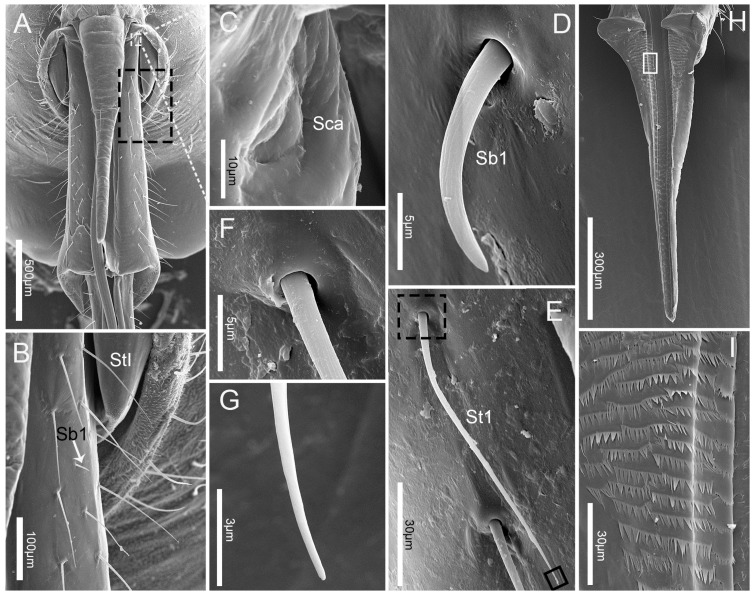
SEM of first labial segment and labrum of *C.nigrescens*. (**A**) Dorsal view of the first labial segment and labrum; (**B**) enlarged view of outlined box in (**A**), showing sensilla trichodea I (St1) and sensilla basiconica I (Sb1); (**C**) enlarged view of sensillum campaniformium (Sca); (**D**) sensillum basiconicum I (Sb1); (**E**) sensillum trichodeum I (St1); (**F**), (**G**) enlarged view of outlined box in (**E**), showing the base and tip of sensillum trichodeum I (St1), respectively; (**H**) epipharynx; (**I**) enlarged view of epipharynx; St 1, sensilla trichodea I; Sca, sensilla campaniformia; Sb1, sensilla basiconica I.

The elongated conical labrum (Lm) attaches to the anterior margin of the anteclypeus (Figure 1A,B, Figure 2A). It is wide proximally and gradually narrowed to the apex with its lateral margins bent around ventrally and its surface strongly plicated. The labrum is closely adpressed over the first labial segment and partly embedded in the labial groove. The dorsal side of the labrum bears a pair of sensilla trichodea I (St1). Sensilla trichodea I (St1) (Figure 2) are the longest, with a slightly grooved wall, a narrowed and rounded tip, and a flexible socket (fs) (Table 2, Figure 2, Figure 3A). Clusters of microtrichia (cp) are arranged in irregular transverse rows on the epipharynx, or ventral area of the labrum (Figure 3H,I).

### 3.3. Labium

The labium (Lb) is long, slender, four segmented, and sub-cylindrical in cross-section, with a proximal and a distal opening. It is normally folded underneath the head when at rest. Its anterior surface is deeply concave to form a longitudinal channel containing the mandibular and maxillary stylets (Figure 1A–C). The four labial segments, from proximal to distal, differ in morphology and size (Table 1, Figure 1A–C). 

**Figure 4:**
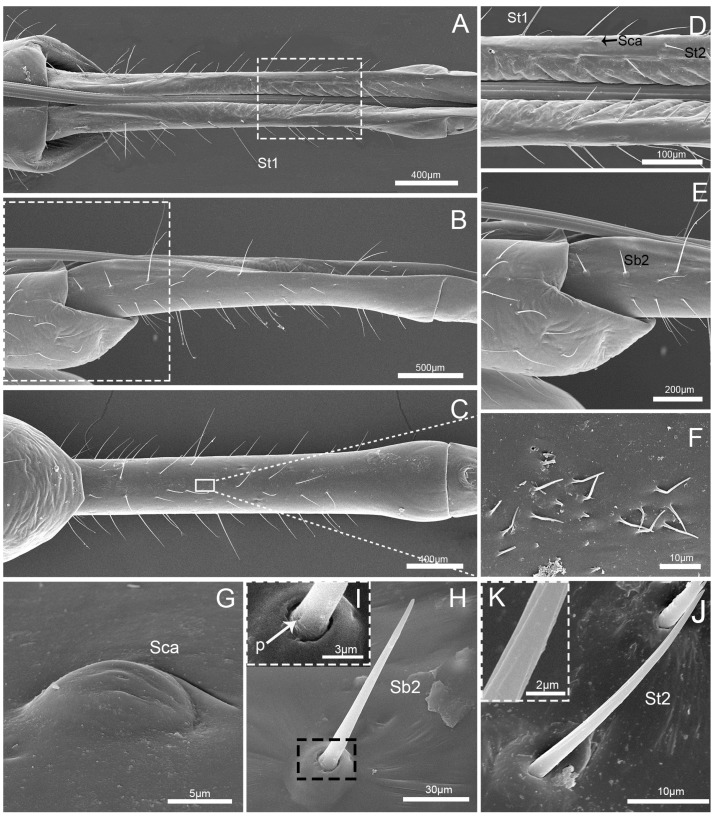
Scanning electron microscopy (SEM) images of the second labial segment. (**A**) Dorsal view; (**B**) lateral view; (**C**) ventral view; (**D**) enlarged view of the box in (**A**); (**E**) enlarged view of the box in (**B**); (**F**) enlarged view of the box in (**C**) showing microtrichia; (**G**) enlarged view of sensilla campaniformia (Sca); (**H**) enlarged view of sensillum basiconicum II (Sb2); (**I**) enlarged view of the box in (**H**), showing the base of sensillum basiconicum II (Sb2); (**J**) enlarged view of sensillum trichodeum II (St 2); and (**K**) enlarged view of the surface of sensillum trichodeum II (St 2). St 1, sensilla trichodea I; St 2, sensilla trichodeaII; Sca, sensilla campaniformia; Sb2, sensilla basiconica II; and p, pore.

The first labial segment (Lb1) is fused with the maxillary plates at the base (Figure 3A). It is broader than the other segments and gradually widens distally with the apex rounded in dorsal/ventral view (Figure 1A). The distal ventral margin of the first labial segment is protruded and completely covers the joint between the two segments dorsally (Figure 3A, Figure 4A,B,E). This structure facilitates an elbowlike bend of the labium between the first and second segment. Three types of sensilla were found on this segment, including a pair of sensilla campaniformia (Sca) that are located at the junction of the head and first segment, many sensilla trichodea I (St1) that are arranged on each side of the labial groove and on the lateral surface and a few on the ventral surface, and several sensilla basiconica I (Sb1) that are arranged on the dorsal surface (Figure 2A,C). St1 are very long and slender (107.1 ± 14.1 μm) and have minute longitudinal grooves in the shaft (Figure 2, Figure 3A,B,E–G). The sensilla basiconica I (Sb1) are short, small, and with a slightly grooved wall and a rounded tip (Table 2; Figure 3D). Sensilla campaniformia (Sca) are small, domelike structures (Table 2; Figure 3C).

The second labial segment (Lb2) is shorter and narrower than the first segment, of uniform width on both sides, and slightly widened at the distal end (Figure 4A–C). Four types of sensilla were found on this segment, including three pairs of sensilla basiconica II (Sb2) that are arranged at the junction of the first and second segment, many sensilla trichodea I (St1) and sensilla trichodea II (St2) interlaced on each side of the labial groove and on the lateral surface and a few on the ventral surface, a pair of sensilla campaniformia (Sca) that are arranged on the dorsal surface, and many microtrichia that are arranged on the ventral surface (Figure 4A–F). St1 and St2 have minute longitudinal grooves in the shaft, but St1 are very long and slender (107.1 ± 14.1 μm) and St2 are short (39.2 ± 11.2 μm). The sensilla basiconica II (Sb2) are short and straight, and with a smooth surface, a rounded tip, a flexible socket, and a single pore (p) that occurs on the basal surface (Table 2; Figure 4H,I). Sensilla campaniformia (Sca) are domelike structures with a single pore (p) (Table 2; Figure 4G).

**Figure 5:**
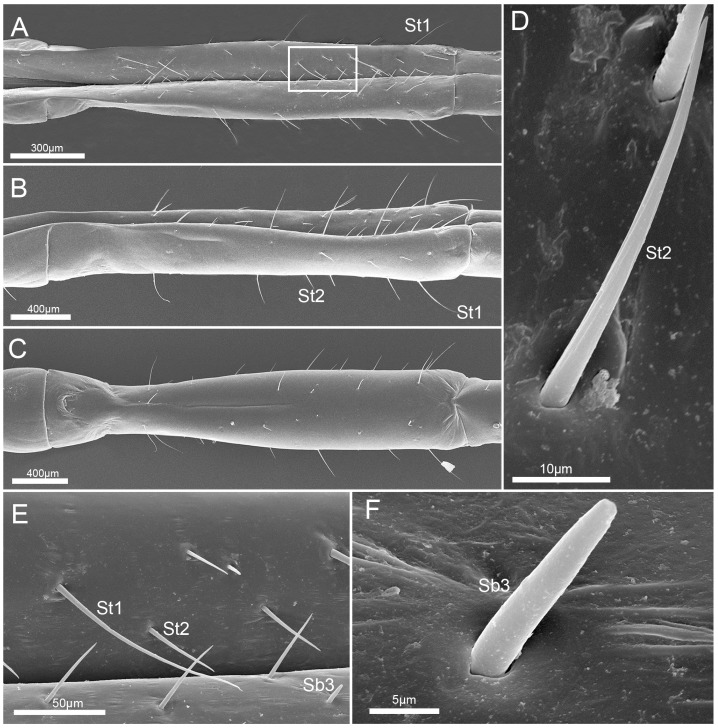
SEM images of the third labial segment. (**A**) Dorsal view; (**B**) lateral view; (**C**) ventral view; (**D**) enlarged view of sensillum trichodeum II (St 2); (**E**) enlarged view of the box in (**A**), showing sensilla trichodea I (St 1), sensilla trichodea II (St 2), and sensilla basiconica III (Sb3); and (**F**) enlarged view of sensillum basiconicum III (Sb3). St 1, sensilla trichodea I; St 2, sensilla trichodea II; and Sb3, sensilla basiconica III.

The third labial segment (Lb3), the shortest of the four segments, is wide at the base, slightly constricted in the basal 1/5, and wide at the apex (Table 1; Figure 5A–C). Many sensilla trichodea I (St1) and sensilla trichodea II (St2) are interlaced on surface (Figure 5A–E). Sensilla basiconica III (Sb3) are sparsely arranged on the dorsal surface (Figure 5E,F). They are short, straight, and have a smooth surface and a rounded tip (Table 2; Figure 5F).

**Figure 6:**
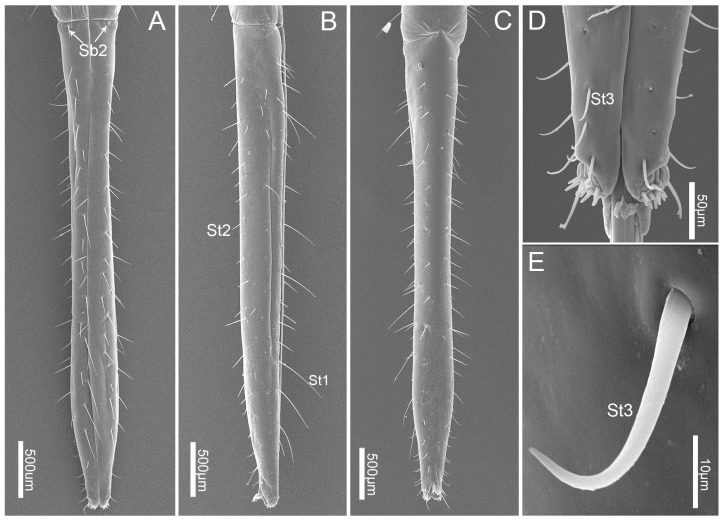
SEM images of the fourth labial segment. (**A**) Dorsal view; (**B**) lateral view; (**C**) ventral view; (**D**) apex of fourth labial segment from dorsal view; and (**E**) enlarged view of sensillum trichodeum III (St 3). St 1, sensilla trichodea I; St 2, sensilla trichodea II; St 3, sensilla trichodea III; St 4, sensilla trichodea IV; and Sb2, sensilla basiconica II.

The fourth, or distal, labial segment (Lb4) is longer than the second and third segment (Table 1). It is conical in shape and tapered distally (Figure 6A–C). A large number of sensilla trichodea I (St1) and sensilla trichodea II (St2) are interlaced on the surface (Figure 6A–C). Two sensilla basiconica II (Sb2) are present on the junction of the third and fourth segments (Figure 6A). They are quite straight, with smooth surfaces, projecting out from a convex round base and almost perpendicular to the surface. Several sensilla trichodea III (St3) are located on the fourth segment surface near the apical 1/6, are short, have a smooth surface, are slightly curved in the apical half, and project out from a convex round base (Table 2; Figure 3D,E). 

**Figure 7:**
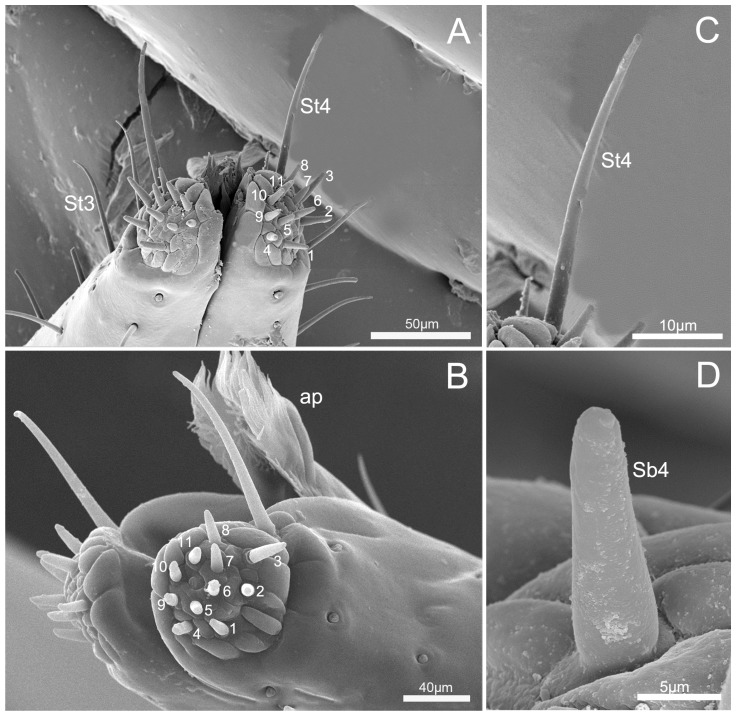
SEM images the tip of labium of *C. nigrescens.* (**A**) Vertical view of the tip of labium showing 11 sensilla basiconca IV (Sb 4), sensilla trichodea III (St 3), and sensilla trichodea IV (St 4); (**B**) half view of labial tip showing 11 sensilla basiconica IV (Sb 4) and apical plate (ap); (**C**) enlarged view of sensillum trichodeum IV (St 4); and (**D**) enlarged view of sensillum trichodeum IV (Sb 4).

The labial tip is tripartite, distinctly divided into two lateral lobes and an apical plate (ap) (Figure 7A,B). Sensilla are symmetrically arranged on two lateral lobes and form two sensory fields (Figure 7A) including two types of sensilla: short stout sensilla basiconica IV (Sb4, no. 1–11) and long hair-like sensilla trichodea IV (St4). Eleven sensilla basiconica are the shortest, located at the center of each lobe and arranged in a central row (Table 2; Figure 7A,B,D). A pair of long sensilla trichodea IV (St4) are located on each side of the apical lobes of the ventral surface (Figure 7A). Sensilla trichodea IV (St4) are hair-like with blunt tips and a smooth surface, and are slightly curved (Table 2 and Table 3; Figure 7C).

### 3.4. Stylet Fascicle

**Figure 8:**
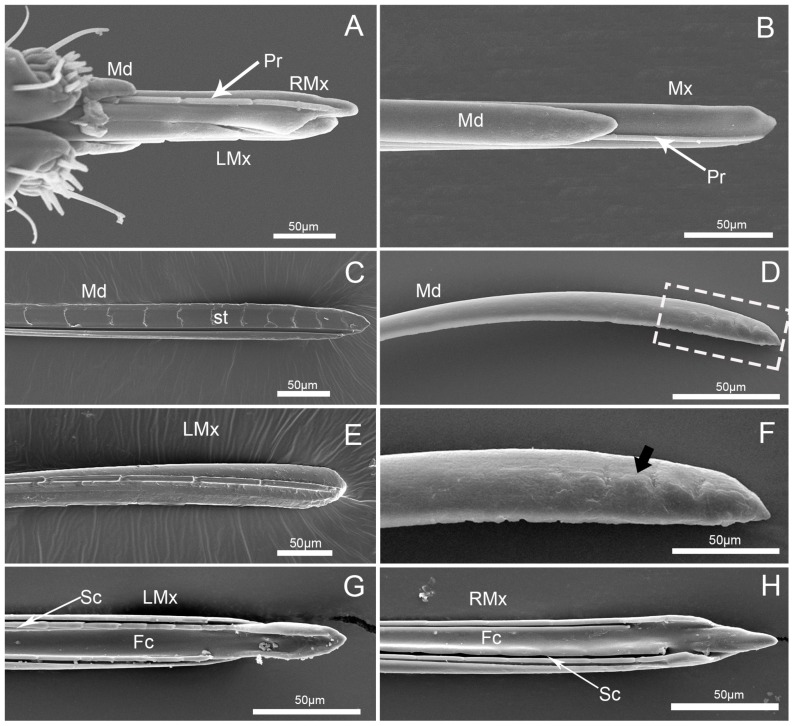
SEM images of stylet of *C.nigrescens*. (**A**) Enlarged anterior view of apex of stylet fascicle; (**B**) enlarged view of stylet fascicle; (**C**) interior view of mandibular stylets (Md); (**D**) external view of mandibular stylets (Md); (**E**) external view of left maxillary stylet (LMx); (**F**) enlarged view of the box in (**D**), showing notches (arrow); (**G**) interior view of left maxillary stylet (LMx); and (**H**) interior view of right maxillary stylet (RMx); Fc, food canal; Pr, process; Sc, salivary canal; Sr, serrate ridges; and st, squamous texture.

The needle-like stylet fascicle is slender and composed of two separated mandibular stylets (Md) and two interlocked maxillary stylets (Mx) (Figure 8A,B). The maxillary stylets (Mx) are slightly longer than the mandibular stylets (Md) (Table 1; Figure 8A). The mandibular stylets ensheath the maxillary stylets laterally.

**Figure 9:**
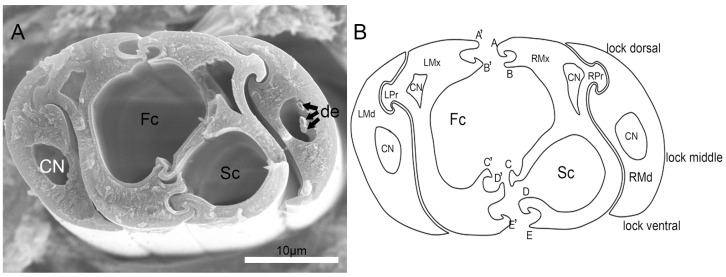
Cross-section of stylet of *C.nigrescens*. (**A**) Cross-section of stylet fascicle; (**B**) diagram of cross-section of stylet fascicle. A, hooked upper right process of the dorsal lock; A’, straight upper left process of the dorsal lock; B, straight lower right process of the dorsal lock; B’, hooked lower left process of the dorsal lock; C, T-shaped right process of the middle lock; C’, hooked upper left process of the middle lock; CN, nerve canal; D, hooked upper right process of the ventral lock; D’, hooked lower right process of the middle lock; de, dendrite; E, straight lower right process of the ventral lock; E’, hooked lower left process of the ventral lock; Fc, food canal; LMd, left mandibular stylet; LMx, left maxillary stylet; LPr, left process of the maxilla; RMd, right mandibular stylet; RMx, right maxillary stylet; RPr, right process of the maxilla; and Sc, salivary canal.

The two mandibular stylets (Md) are mirror images of each other. They are crescent-shaped in cross-section, and concave internally to form a groove for positioning of the maxillary stylets (Figure 9A,B). The outer surface of the mandibular tip has about 6–8 obscure lateral notches (Figure 8D,F), which are irregularly transverse. The inner surface of the mandibular stylet has a regular series of transverse squamous textures (st) (Figure 8C), different from the venter of the longitudinal groove (Figure 8C), which fits to the rim of the adjacent maxilla stylet, causing considerable friction and forcing the stylet to curve inward during probing of plant tissue.

The maxillary stylets (Mx) are interlocked by a hooklike hinge, which is asymmetrical (Figure 9A,B). The external and inner surfaces are both smooth, but equipped with a series of ridges and grooves internally (Figure 8E,G,H) and an external longitudinal process (pr) that engage grooves in the mandibular stylets (Figure 8A,B,E). The ends of the external surfaces of the maxillary stylets are flattened (Figure 8B).

Cross-sections through the stylet bundle (interlocked maxillae and mandibles) in this mirid species (Figure 9A) show that the bundle is distinctly depressed, and wider than tall. The two maxillary stylets have a three-part inner locking system, including five processes on the arm of the right maxilla and five on the left (Figure 9B). On the right maxilla, the dorsal and ventral locks are formed by a hooked process and a straight process. The middle lock is formed by only a T-shaped process (Figure 9B). On the left maxilla, the dorsal lock is formed by two processes: a straight upper one and a hooked lower one. The middle lock is formed by two hooked processes (Figure 9B). The ventral lock is formed by only a hooked process. The left and right mandibular stylets are mirror images of each other; they are placed laterally with respect to the maxillae and connected to the latter by a one-lock system. Within each mandibular stylet there are three dendrites (Figure 9B). There are two internal ducts (Figure 9A,B): the food canal (Fc) and the salivary canal (Sc). The food canal is separated from the salivary canal by apposed ridges on the two contiguous maxillary stylets (Figure 9A,B). The central food canal (Fc) is usually oval in cross-section due to the asymmetrical concave inner walls of the two stylets. The diameter of the food canal is greater (9.7 ± 0.7 μm, *n =* 5) than that of the salivary canal (5.9 ± 0.3 μm, *n =* 5). The food and salivary canals end just proximal to the stylet tip, where the salivary canal joins the food canal.

## 4. Discussion

The different feeding habits of true bugs usually correspond to differences in mouthpart structures [7,17]. Most mirids are plant feeders, but some species are scavengers or facultative predators [18]. Morphological comparisons of both mandibular and maxillary stylets revealed differences among mirids with different feeding preferences [7,10,36]. This is the first detailed description of the mouthparts of *C. nigrescens*, including various sensilla and their arrangement on the labium. Compared with other mirids (Table 3), including carnivorous *Deraeocoris nigritulus* (Uhler) [10] and *Deraeocoris nebulosus* (Uhler) [11], phytophagous *Deraeocoris oliveceus* (Fabricius) [7], *Lygus lineolaris* (Palisot de Beauvois) [23,24], *Lygus rugulipennis* (Poppius) [25], *Notostira erratica* (L.) [26], *Dicyphus hesperus* Knight [27], *Isometopus intrusus* (Herrich-Schaeffer) [7], and *Lygus pabulinus* (L.) [7,28], the mouthparts of *C. nigrescens* adults appear to display a number of traits associated with phytophagy. 

Various traits of the stylets, including the shape and dentition of the tips and size of the food canal, have been studied previously in several heteropterans [7,21,37]. Compared to sensory receptors on the labium, the mirid stylets have been poorly described. The mirid mandibular tip has a moderate number of lateral notches or serrations, which extend somewhat transversely in both phytophagous and carnivorous species [7,10,11,27]. Similar structure is also found in other heteropteran species but the numbers and patterns of teeth are different [7,21,27,38,39,40]. We also observed about 6–8 notches on the dorsal margin of the convex external surface. Because mirids do not produce a salivary flange, the apical serrations on the mandibular stylets are thought to be adaptations for anchoring the stylets in tissues below the outer layer of the prey or host plant and producing a fulcrum for movement of the maxillary stylets [7,20,41]. However, notches in *C. nigrescens* are indistinct and differ from the deep serrations in the mandibular stylets of predatory species [10,11,27]. A series of squamous textures (st) regularly present on the inner surface of the mandibular stylet, which is similar to those found in several other heteropterans [7,42]. Cobben [7] indicated that the orientation of this parallel groove is such that the forward thrust of one mandible will cause considerable friction against the outer surface of the adjacent maxillary stylet, contributing to its inward bend.

The inner surface of the right maxillary stylet of heteropterans is often moderately serrated in predatory families but smooth in strictly phytophagous families, with the Miridae showing an intermediate condition [7]. By comparing maxillary stylets from strictly zoophagous to strictly phytophagous mirids, Boyd [43] suggested that the serrations of the right maxillary stylets are deeper for predators than in herbivores. Moreover, the right maxillary stylet has two rows of at least six recurved barbs on the inner surface in the predacious *D. nebulosus* [11], and there are two rows of at least seven strongly recurved teeth in front of at least three weakly recurved teeth on the inner surface in predacious *D. nigritulus* [10]. In our study, the right maxillary stylet has no teeth on the inner surface of the right maxillary stylet. Cobben [7] suggested that the plant-feeding Miridae originated from carnivorous forerunners. After the shift to phytophagy, the spines or teeth on the stylets may have been no longer useful or may even have hindered piercing-sucking of plant tissues, while the requirements for stability and saliva were strengthened, resulting in reduction and loss of these structures [36]. In *C. nigrescens*, the maxillary stylets (Mx) are smooth externally but equipped with a longitudinal ridge that engages grooves in the mandibular stylets, causing it to curve inward during probing of plant tissue [7,13,28,42].

Brożek and Herczek [13] have studied the interlocking mechanism of maxillae and mandibles in Heteroptera, previously identifying three locks between the maxillae, i.e., dorsal, middle, and ventral. We observed the same internal structure in *C. nigrescens* mouthparts. Additionally, as found by Brożek and Herczek [13] in other Heteroptera, the food canal of *C. nigrescens* is oval and the salivary duct smaller than the food canal, which is circular in cross-section. There are also five processes on the right and left maxilla, as found in their study of representatives of 15 species of Miridae. No differences were noted between carnivorous and phytophagous mirids in structure of stylet in cross-section.

The labium of hemipterans plays the important roles of not only receiving the maxillary and mandibular stylets but also recognizing host clues using the sensory structures present on its surface [44]. Four types of sensilla on the tip and surface were observed on the labium of *C. nigrescens*. The most abundant sensilla on the labium are sensilla trichodea, which have no pores and are therefore considered to be mechanoreceptive [20]. Sensilla trichodea IV with flexible sockets in the subapical region of the labium in this species appear to be identical to those of other heteropterans, in which they are apparently mechanosensilla and may play a role in sensing the feeding sites [23]. The pair of sensilla basiconica II on the junction between the first and second segment, and the third and fourth segment, which also occur in *Cacopsylla chinensis* (Yang et Li), *Lycorma delicatula* (White), *Eriosoma lanigerum* (Hausmann), and *Pyrrhocoris sibiricus* Kuschakevich [45,46,47], may function as proprioceptors to perceive the degree of flexion of the joint [2,45]. Two pairs of sensilla campaniformia are arranged bilaterally at the distal part of the anterior surface of the second labial segment of *C. nigrescens*. Similar sensilla in Peiratinae (Reduviidae) have been shown to have a proprioreceptory function [48], and such mechanosensory sensilla also occur in phytophagous Pentatominae and Pyrrhocoridae [35], and predatory Asopinae (Pentatomidae) [17]. In mirids, such sensilla probably act as proprioceptors responding to the stresses arising from the movement of the labium. 

Eleven pairs of sensilla basiconica IV are arranged bilaterally and symmetrically around the opening at the tip of the labium in two sensory fields of *C. nigrescens* (Figure 7), as reported in the mirids *L. pabulinus* [28], *L. lineolaris* [23,24] and *L. rugulipennis*, the pentatomid *N. viridula* L. [8], and the blissid *Blissus leucopterus leucopterus* (Say) [49]. Awati [28] described the labial tip sensilla on *Lygus pabulinus* as bristles that function as sensory hairs. Avé et al. [23] reported that the rostral tip of the plant bug *L. lineolaris* contains two sensory fields each containing 11 sensilla basiconica. Hatfield and Frazier [24] also observed that the tripartite apex of the labium possesses two fields of 11 uniporous peg sensilla; nonporous hair sensilla; and the apical plate, which is a non-innervated structure possessing terminal cuticular projections in the plant bug *L. lineolaris*. Romani et al. [25] also reported that the tip of the labium of *L. rugulipennis* females presents two lateral lobes bearing 11 sensilla basiconica, which are innervated by 3–6 sensory neurons. They also reported that morphological evidence supports previously reported physiological evidence that the uniporous peg sensilla have a chemosensory function [24,25]. A detailed comparison of labial tip sensilla of *C. nigrescens* with those of other plant bugs suggests that the arrangement of these sensilla (22 sensilla basiconica and 2 sensilla trichodea) in mirid species is mostly stable [23,24,25]. Cobben [7] suggested that the shape and arrangement of sensilla on the tip are not correlated with carnivorous or phytophagous feeding habits, and in Miridae and in Pentatomorpha there appears to be a tendency for bugs to have a more regular double row of sensilla. Generally, the positions and arrangement of sensilla were similar among the studied phytophagous species, and sensilla basiconica on the apical sensorial region probably have contact chemoreceptor (gustatory functions). In mirids, such sensilla may help guide the mouthparts during penetration of the host tissue in order to find the most appropriate feeding site [23,24,38,42,50,51].

The feeding mechanism may be inferred from the mouthpart morphology of insects. Based on its structure, the labrum is probably not moveable in *C. nigrescens* Liu et Wang. First, plant-feeding mirids orient toward potential hosts by visual and olfactory cues. After arriving on a potential host, mirids stretch out the labial segments and begin exploring the surface, periodically tapping with the labium. At the same time, mirids secrete copious amounts of saliva containing digestive enzymes to test host suitability, which are dabbed on the plant surface and sucked up to internal organs. After locating a feeding site using sensilla, the labium bends and the stylet fascicle stretches out. The sharp end and notches of the mandibular stylets pierce the plant cuticle and epidermis while saliva is secreted. The left and right mandibular stylets penetrate alternately and anchor the stylets in place in the plant tissue, followed by protraction of the maxillary stylets. The maxillary stylets pierce the tissue to the required depth aided by enzymes in the watery saliva. Through a series of backward and forward movements of the stylets, the sheath is lengthened until the desired feeding site has been reached. During feeding, the stylets are inserted intercellularly or intracellularly, lacerating mesophyll cells during active movement. As the stylets progress through the plant tissue, mirids secrete more of the viscous saliva and inject salivary pectinase into the plant tissue to macerate the cells. Then, the maxillary stylets transport the contents of cells with a more or less copious flow of saliva to the mouth by a relatively air-tight food canal [18,20,44].

## 5. Conclusions

This study provides the first detailed observations on the mouthparts of the phytophagus mirid bug, *C. nigrescens*, including some details, special structures of the labium, stylet bundles, and different types of labial sensilla. Overall, the mouthparts of this species do not strongly differ from those of other studied plant-feeding species of Miridae, indicating that the mouthpart structure of this family, including arrangement of sensilla, is highly conservative. The indistinct notches of the mandibular stylet and smooth inner surface of the right maxillary stylets indicate that the mouthparts of this species are suited primarily for phytophagy. 

## Figures and Tables

**Table 1:** Measurements of labrum, labium, and stylets (mean ± SE) of male and female obtained from scanning electron microscopy. N = sample size. Lb-sg1, the first segment of labium; Lb-sg2, the second segment of labium; Lb-sg3, the third segment of labium; Lb-sg4, the fourth segment of labium; Lm, labrum; Md, mandibular stylet; and Mx, maxillary stylet.

Gender	Position	Length (μm)	Width (μm)	Height (μm)	N
Female	Lb	3798.4 ± 37.8	-	-	3
Male	Lm	705.6 ± 48.9	230.0 ± 3.5	-	5
	Lb-sg1	1012.6 ± 30.3	224.8 ± 17.1	128.7 ± 22.4	12
	Lb-sg2	886.9 ± 38.3	154.3 ± 7.5	121.5 ± 5.8	12
	Lb-sg3	796.6 ± 52.8	121.8 ± 8.5	126.0 ± 7.8	12
	Lb-sg4	1004.3 ± 50.4	136.4 ± 8.2	104.3 ± 5.6	12
	Md	3929.5 ± 193.2	16.2 ± 0.5	-	11
	Mx	4123.4 ± 107.8	18.0 ± 0.6	-	11

**Table 2:** Morphometric data (mean ± SE) for various sensilla of adult C. nigrescens. *N* = sample size. Lb-sg1, 2, 3, 4, the first, second, third, and fourth segment of labium; Lm, labrum; Sb1–4, sensilla basiconica I–IV; Sca, sensilla campaniformia; SF, sensory field on the labial tip; and St1–4, sensilla trichodea I–IV.

Sensilla Type	Distribution	Length (μm)	Basal Diameter (μm)	*N*
St1	Lm, Lb-sg1, 2, 3, 4	107.1 ± 14.1	2.8 ± 0.3	20
St2	Lb-sg1, 2, 3, 4	39.2 ± 11.2	2.5 ± 0.4	20
St3	Lb-sg4	22.9 ± 4.5	1.8 ± 0.3	20
St4	Lb-sg4	33.2 ± 2.8	2.4 ± 0.2	15
Sb1	Lb-sg1, 2	13.4 ± 3.0	1.9 ± 0.2	15
Sb2	Lb-sg2, 4	41.2 ± 8.9	3.1 ± 0.4	15
Sb3	Lb-sg3	8.9 ± 1.20	1.9 ± 0.4	15
Sb4	SF	7.1 ± 1.3	2.1 ± 0.2	15
Sca	Lb-sg1, 2	-	9.8 ± 1.1	15

**Table 3:** Main features of mouthparts of Miridae. * Characters in parentheses were summarized from figures in Cobben 1978 [7].

Species Name	Labrum	Labium	The Number of Basiconica Sensilla of Labium tip	The Number of Sensilla Type	Apical Plate	The Number of Barbs on the Right Maxillary Stylet Tips	Squamous Texture on Mandibular Stylet	The Number of Teech on the Distal Mandibular Stylet	References
*Lygus lineolari*	unreported	unreported	11 pairs	unreported	√	unreported	unreported	unreported	Hatfield and Frazier 1980 [24]; Avé et al. 1978 [23]
*Lygus pabulinus*	tapers to a point, the base being broader	4-segment	unreported	unreported	√	(right stylet being more roughly armoured than the left one)	√	7–9 recurved hooks	Cobben 1978 [7] * Awati 1914 [28]
*Lygus rugulipennis*	unreported	4-segment	11-12 pairs	unreported	√	unreported	unreported	(cuticular teeth)	Romani et al. 2005 [25]
*Isometopus intrusus*	unreported	unreported	unreported	unreported	√	(recurved barbs)	√	(lateral notches)	Cobben 1978 [7] *
*Dicyphus hesperus*	unreported	unreported	unreported	unreported	unreported	unreported	unreported	(Serrations on the lateral margins)	Roitberg et al. 2005 [27]
*Deraeocoris oliveceus*	unreported	unreported	unreported	unreported	√	(two rows strongly teeth)	√	(lateral notches)	Cobben 1978 [7] *
*Cheilocapsus nigrescens*	elongated conical	4-segment	11 pairs	10	√	no	√	about 6–8 obscure lateral notches	This study
*Deraeocoris nebulosus*	unreported	unreported	unreported	unreported	unreported	two rows of at least six recurved barbs	unreported	unreported	Boyd et al. 2002 [11]
*Deraeocoris nigritulus*	unreported	unreported	unreported	unreported	unreported	two rows of at least seven strongly recurved teeth	unreported	unreported	Boyd 2003 [10]
